# The relationship between myodural bridge, atrophy and hyperplasia of the suboccipital musculature, and cerebrospinal fluid dynamics

**DOI:** 10.1038/s41598-023-45820-x

**Published:** 2023-11-02

**Authors:** Heng Yang, Xiao-Song Wei, Jin Gong, Xue-Mei Du, Hong-Bo Feng, Chang Su, Campbell Gilmore, Chen Yue, Sheng-Bo Yu, Chan Li, Hong-Jin Sui

**Affiliations:** 1 Department of Anatomy, Dalian Medical University, Dalian, Liaoning, China; 2 Department of Nuclear Medicine, The First Affiliated Hospital of Dalian Medical University, Dalian, Liaoning, China; 3 The First Affiliated Hospital of Dalian Medical University, Dalian, Liaoning, China; 4 Dr Grays Hospital, NHS Grampian, Elgin, Scotland; 5 Department of Gynecology ands Obstetrics, Affiliated Zhongshan Hospital of Dalian University, Dalian, Liaoning, China

**Keywords:** Musculoskeletal system, Nervous system

## Abstract

The Myodural Bridge (MDB) is a physiological structure that is highly conserved in mammals and many of other tetrapods. It connects the suboccipital muscles to the cervical spinal dura mater (SDM) and transmits the tensile forces generated by the suboccipital muscles to the SDM. Consequently, the MDB has broader physiological potentials than just fixing the SDM. It has been proposed that MDB significantly contributes to the dynamics of cerebrospinal fluid (CSF) movements. Animal models of suboccipital muscle atrophy and hyperplasia were established utilizing local injection of BTX-A and ACE-031. In contrast, animal models with surgical severance of suboccipital muscles, and without any surgical operation were set as two types of negative control groups. CSF secretion and reabsorption rates were then measured for subsequent analysis. Our findings demonstrated a significant increase in CSF secretion rate in rats with the hyperplasia model, while there was a significant decrease in rats with the atrophy and severance groups. We observed an increase in CSF reabsorption rate in both the atrophy and hyperplasia groups, but no significant change was observed in the severance group. Additionally, our immunohistochemistry results revealed no significant change in the protein level of six selected choroid plexus-CSF-related proteins among all these groups. Therefore, it was indicated that alteration of MDB-transmitted tensile force resulted in changes of CSF secretion and reabsorption rates, suggesting the potential role that MDB may play during CSF circulation. This provides a unique research insight into CSF dynamics.

## Introduction

Cerebrospinal fluid (CSF) is a clear liquid that is found in the ventricles and subarachnoid space of the central nervous system (CNS) and is the major extracellular fluid of the CNS^1,2^. CSF plays an important role in a number of physiological processes. As a buoyant medium, CSF provides mechanical support and reduces the effective weight of the brain by allowing it to float^3^. CSF also helps to cushion the brain and spinal cord from pressure, especially during trauma^3^. Additionally, CSF is responsible for nourishing neural tissue and removing metabolic waste products from the CNS^2,3^. Together with intracranial vessels, CSF also helps to maintain homeostasis in the intracranial environment^4^. Thus, the regulation of CSF circulation is critical for CNS health^5^. CSF circulation involves the production, drainage, and reabsorption of fluid within the CNS^1,5^. Compare to the CSF in spinal tube, ventricular CSF features more clinical implications. The cellular lining of the ventricle both can produce and is responsive to CSF. Anomalies of the CSF/ventricular system serve as diagnostics and may cause CNS disorders, further highlighting their importance^6^. While the flow direction, velocity, and quantity of CSF have been studied, the precise mechanism of CSF circulation remains unclear^7–10^. However, it is thought to be influenced by factors such as arterial pulsations, respiratory movements, body position, intracranial blood circulation, and the myodural bridge (MDB)^11–14^.

The fibrous structure known as the MDB connects the suboccipital musculature to the cervical spinal dura mater (SDM) via the atlantooccipital and atlantoaxial interspace^15,16^. Comparative anatomical studies have revealed that the MDB is an evolutionarily conserved structure found in both terrestrial and marine mammals, reptiles, and birds^17–20^. The MDB's shape, location, fiber properties, and bridging characteristic have led to several speculated functions, including preventing in-folding of the SDM during head extension, transmitting proprioception, maintaining the integrity of the subarachnoid space, and affecting CSF circulation^21–23^. To better understand the MDB's functions, Zheng et al^24^ proposed the concept of the MDB complex (MDBC), an integrated functional unit involving several muscles, ligaments, and MDB fibers, in this study, the authors suggested that the CSF circulation could be changed by cervical body motion after the status of MDBC has been changed.

However, it has been argued that CSF dynamics is influenced in quadrupeds with a minor exposition to hydrostatic forces by their more horizontally oriented aqueduct, and thus body motions in quadrupeds may be more critical regulators to CSF dynamics compare to humans^25^. And for humans, the aqueduct is at more of a vertical position, and thus the humans may require more powers to equilibrate CSF against hydrostatic forces; even so, up to date, the direct experimental evidence showing hydrostatic force is the key factor for CSF circulation remains missing.

However, previous studies have demonstrated that in humans, head rotation and nodding can alter the volume, velocity, and pressure of CSF flow^14,26^. Furthermore, experiments conducted on dogs have suggested that in humans, MDB and body motions have strong potentials to play a role in CSF circulation through controlling the suboccipital muscles^23^. Moreover, our previous study revealed that hyperplasia of suboccipital muscles lead to increased intracranial pressure in rats^27^, suggesting the body motion, MDBC dysfunction, and pressure changes inside the CSF circulation pathways are correlated to each other, together form a dynamic equilibrium. More to the point, patients with Chiary I malformation exhibited hindered CSF dynamics^28^, suggested that without changes in upright position or hydrostatic forces of human CSF, a local malformation or dysfunction at cervical level could be enough to unbalance the CSF dynamics or circulation in humans. These collectively suggested that not only the hydrostatic forces, MDBC is highly possible to be one of the key regulatory factors in CSF dynamics of humans as well. As this result, although these studies only examined or implied the transient effects of MDB on CSF circulation, it is indicative that a quadrupeds animal model with MDBC dysfunction is capable to be a human mimicry.

It is why the power system of human CSF is still under debate. We in all time believe that humans need more against hydrostatic forces, but at the same time we do not believe the hydrostatic forces plays the key role in CSF dynamics. It must be regulated by multiple factors.

BTX-A is a bacterial exotoxin that targets the neuromuscular junction, inhibiting the release of acetylcholine from nerve terminals, which can cause muscle atrophy^29^. BTX-A injections have been used for aesthetic treatments such as wrinkle reduction, as well as for treating muscle spasticity and hypertrophy^30^. Animals treated with BTX-A exhibited significantly reduced muscle mass and corresponding decreases in muscle strength^31^. ACE-031 is a specific inhibitor of myostatin, also known as GDF-8, a secreted protein that regulates muscle size and function by suppressing myocyte proliferation and differentiation^32,33^. Natural or artificially induced myostatin mutations have been shown to cause marked muscle hypertrophy, resulting from both muscle cell hyperplasia and hypertrophy^34,35^. ACE-031 binds competitively to myostatin proteins, antagonizes the normal myostatin receptor ACTVIIb, and blocks the downstream signaling pathway of myostatin, resulting in a physiological dysfunction of myostatin proteins^33^. A previous study has verified that myostatin local blockage with ACE-031 can increase muscle strength^27^.

In this study, the researchers injected BTX-A and ACE-031 into rats' suboccipital muscles to create suboccipital muscle atrophy (BTX-A group) and hyperplasia (MSTN group) animal models, respectively. For the negative control, the researchers established a suboccipital muscles severed group (SEV group) to surgically eliminate the physiological functions of the suboccipital muscles. Meanwhile, another group without any surgical operation was established as a wild-type control (CTL group). The researchers then measured the secretion rate of CSF in live rats by blocking the aqueduct of Sylvius and inserting a glass capillary into the lateral ventricle^36,37^. The rate of CSF secretion was taken as the rate of CSF formation (V_f_)^38^. The researchers also selected six CP-CSF-related proteins to study the secretion mechanism of CSF^39–44^. Over last two decades, studies showed that the olfactory lymphatic pathway seemed the main way of CSF absorption, rather than arachnoid villus^45–47^. To evaluate the rate of CSF reabsorption (V_a_), the researchers used SPECT/CT to detect the radioactive intensity of the turbinate area after lateral cerebral ventricle injection of radionuclide. By analyzing both V_f_ and V_a_, the researchers explored the mechanism of how muscle strength alteration could lead to different pulling effects of the MDB and changes in CSF dynamics. The results of the study may support the theory that MDBC could be one of the factors affecting the dynamic circulation of CSF and provide unique insights into the mechanism of CSF dynamics.

## Materials and methods

### Ethics statement

This study adhered strictly to the guidelines outlined in the "Guide for the Care and Use of Laboratory Animals" as presented by the National Institutes of Health. The experimental protocol was approved by the Committee on the Ethics of Animal Experiments at Dalian Medical University. All surgical procedures were conducted using Avertin anesthesia, and all possible measures were taken to minimize any potential discomfort or pain experienced by the animals involved. This study was carried out in compliance with the ARRIVE guidelines.

### Animals

Male rats were utilized in this study, and all experimental rats were obtained from different female rats and raised from 8 weeks of age for subsequent experiments. The animals were housed in a controlled environment with a 12-h light cycle and ad libitum access to standard rodent diet (Medicience Ltd. Jiangsu, China) and water. Four experimental groups were established for this study, consisting of 46 animals randomly divided into MSTN group (n = 13), CTL group (n = 13), BTX-A group (n = 10), and SEV group (n = 10). Prior to and 2 weeks after model establishment, measurements of weight, food intake, and water intake were obtained to evaluate the baseline physiological conditions among the groups. Ethical guidelines were strictly followed in the care and use of the laboratory animals, and the study protocol was approved by the Committee on the Ethics of Animal Experiments of the Dalian Medical University.

### Animal models establishment

To control the surgical interference, a group of animals without any surgical operation was firstly established as a wild-type control in contrast (CTL, n = 10).

**Figure 1:**
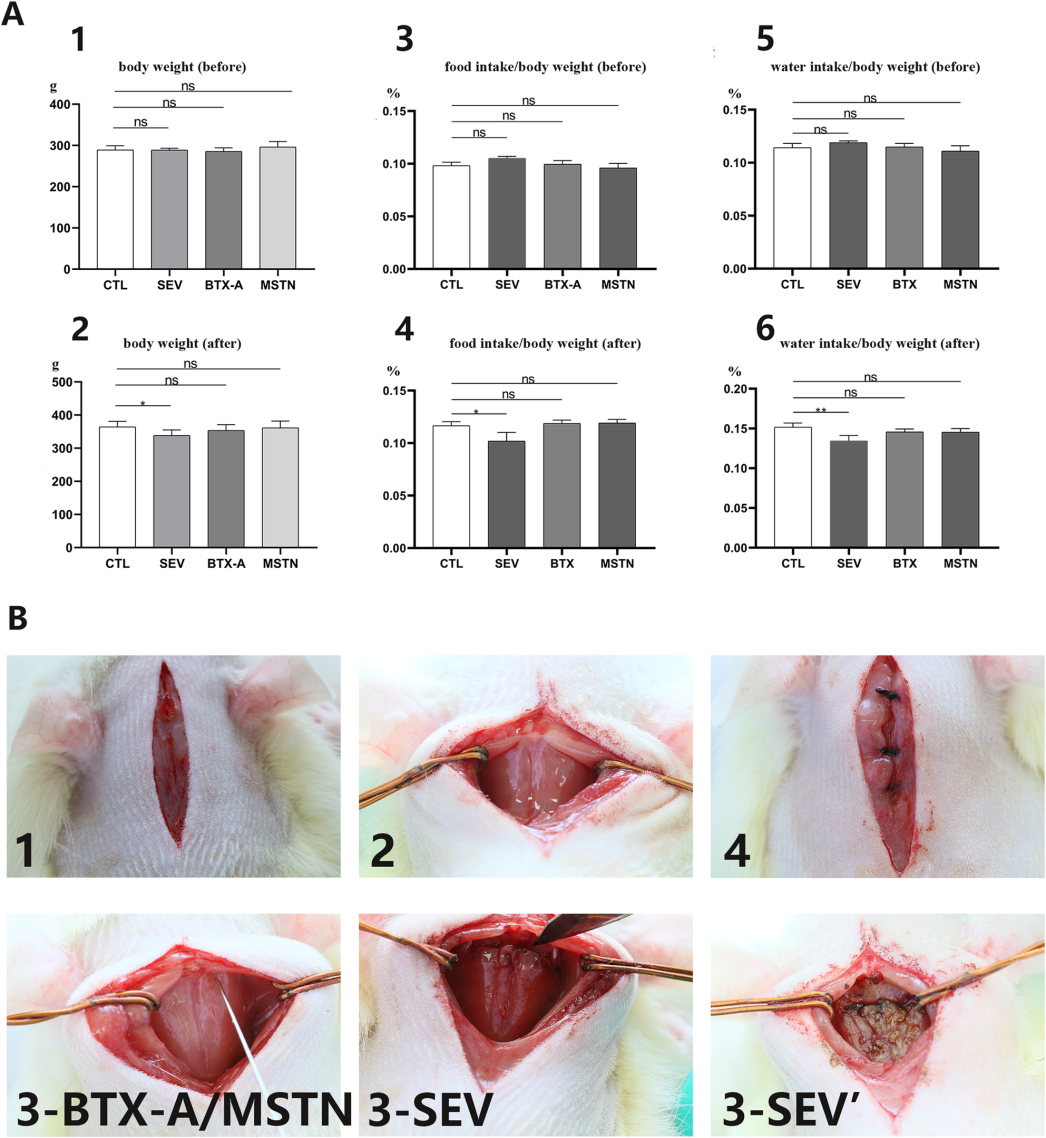
The basic physiological conditions of all groups’ rats and the modeling procedures. (**A**) body weight, food intake/body weight ratio, and water intake/body weight ratio comparison before and 2 weeks after model establishment: 1 and 2. comparison of body weight before and 2 weeks after modeling, 3 and 4. comparison of food intake/body weight ratio before and 2 weeks after modeling, 5 and 6. comparison of water intake/body weight ratio before and 2 weeks after modeling. (**B**) operation method of BTX-A and MSTN groups and SEV group: 1. a median sagittal incision was made on the skin and the superficial layer of muscles, 2. the RCDma muscles was exposed, 3-BTX-A/MSTN. the injection point was located, 3-SEV. the RCDma and RCDmi muscles was cut along the posterior border of the occipital bone, 3-SEV’. The RCDma and RCDmi muscles were removed and then cauterized, 4. suture the superficial layer muscles and skin respectively. CTL, control group; SEV, severance group; BTX-A, BTX-A local injection group; MSTN, ACE-031 local injection group. *, *P* < 0.05; **, *P* < 0.01; ns, no significance.

For the other experimental groups of the present study, prior to the surgery and injection procedure, all rats were fasted overnight. Anaesthesia was induced using a 2.5% Avertin (Sigma-Aldrich, USA) solution administered through intraperitoneal injection. All experimental animals were set on stereotaxic apparatus for the subsequent surgical procedures (RWD68025, RWD, China). A 1.5–2 cm incision was made on the dorsal side of the rat's neck to expose the superficial muscle layer until the midline was visible (Fig. 1 B1). The superficial muscle was then incised along the midline to expose the RCDma (Fig. 1 B2). In the suboccipital muscles severed group of rats (SEV, n = 10), the RCDma and RCDmi were cut along the posterior border of the occipital bone with a scalpel until the dura was visible (Fig. 1 B3-SEV). The RCDma and RCDmi were then separated and taken out along the lateral border of the muscles, and the severed ends of the muscles were cauterized with an alternating current-operated bipolar coagulation device to stop the bleeding and prevent re-connecting to the occiput (Fig. 1 B3-SEV’). The superficial muscles and skin were sutured separately to complete the procedure (Fig. 1 B4). For the BTX-A and ACE-031 local injection group of rats (BTX-A, n = 10; MSTN, n = 13), the entry point was 2 mm below the inferior margin of the occiput, and 5 mm to the left and right of the midline. A 25 μl micro syringe with a calibre of 0.22 mm (32G) was used for all the injections. The angle of the injections was made perpendicular to the inferior surface of the occiput (Fig. 1 B3-BTX-A/MSTN). Once the pinhead touched the posterior edge of the occiput, it was withdrawn by 0.5 mm to ensure it was located in the RCDmi, while it was withdrawn by 5 mm to be in the RCDma. Each rat was injected on both sides of the RCDmi and RCDma. Every injection was performed at a constant speed at 1μ/sec during the entire process. In the BTX-A group, 5 μl BTX-A (Lanzhou Institute of Biological Products Co., LTD, China) was injected at a concentration of 0.05U/μl into each muscle, totalling 20 μl per rat. Similarly, in the MSTN group, a total of 20 μl ACE-031 (Crystal Chemical Inc., IL, USA) was injected at a concentration of 50 ng/μl into each muscle. Following the injection procedure, the superficial muscles and skin were sutured separately to complete the operation (Fig. 1 B4).

### CSF secretion rate measurement

Two weeks after model establishment, the CSF secretion rate was quantified in accordance with the modified method described by Karimy et al^36^ and Liu et al^37^. Rats were anesthetized with avertin and then fixed onto a stereotaxic apparatus (RWD68025, RWD, China). A small hole, approximately 0.6 mm in diameter, was drilled above the right lateral ventricle using a dental drill. The coordinates for the hole (insertion point), relative to bregma, were x = 1.5 mm and y = 0.5 mm (Fig. 3B). Following the removal of the incisor bar, the rat's head was rotated 90° on the ear bar, with its nose oriented vertically downwards. The superficial muscles were dissected to expose the skin, and a trocar was inserted into the fourth ventricle through the posterior atlanto-occipital membrane. The needle core was removed, and clear CSF appeared in the sterile elastic catheter (PE-20). The catheter was advanced 5 mm deeper to enter the fourth ventricle, following which sterile molecular-grade mineral oil (100 µl; Sigma-Aldrich) was injected into the fourth ventricle to obstruct the circulation of CSF in the aqueduct of Sylvius. To avoid any blockages caused by brain parenchyma, a first glass capillary (OD, 0.6 mm; ID, 0.5 mm; VitroCom) was inserted 0.6 mm deep into the right lateral ventricle, then taken out. Subsequently, a second glass capillary (with the same dimensions as the first) was inserted along the same path. The amount of CSF flowing into the capillary was measured after a 10-min wait (Fig. 3A). The volume of CSF (V) (Fig. 3D) was calculated using the formula for the volume of a cylinder: V (mm^3^) = π·r^2^·d, where r is the radius of the capillary (0.25 mm) and d is the distance that CSF moves in the capillary. The rate of CSF formation (µl/min) was then calculated from the slope of the volume-time relationship.

Moreover, to assess the efficacy of mineral oil in obstructing the aqueduct of Sylvius, and to confirm the insertion of the catheter reaches the fourth ventricle, before we actually started to test the CSF secretion rates, a blockage test was conducted. Similar to the procedure which was described above, right after the oil injection into the fourth ventricle, the rat's head was positioned horizontally (back to normal position), and 20 μl of eosin was slowly injected at a rate of 5 μl/min using a micro syringe. To ensure proper staining, the syringe was removed after 10 min of injection. The rat's brain was extracted following a standard perfusion process, and sliced horizontally to visualize all ventricles at the same level. The staining of each ventricular wall was observed under real-time conditions, no staining or damage could be seen on any parts of brain tissues; thereby confirmed the efficacy of the oil blockage as well as the positioning of the fourth and the lateral ventricle insertion. All images were captured using a Canon EOS 7D Mark II digital SLR camera (Canon Inc., Tokyo, Japan) (Fig. 3C).

### CSF reabsorption rate measurement

Two weeks after the establishment of the animal models, the CSF reabsorption rate was measured using standard procedures. Specifically, rats were prepared as described previously, up to the point of skull hole drilling and with their heads placed in a horizontal position. A micro syringe containing 15 μl Technetium 99-labeled diethylene-triamine-pentaacetate (99mTc-DTPA), a radioactive tracer, was inserted 0.6 mm deep into the right-side lateral ventricle and injected at a rate of 3 μl/min (see Fig. 5A). The syringe was left in place for an additional 1–2 min after injection to prevent reflux of the tracer and promote its circulation in the CSF. The hole was immediately sealed with bone wax upon removal of the syringe. Subsequently, a SPECT/CT scan (SIEMENS Symbia Intevo 6) was conducted for 25 min (5 frames at 1 frame/3 min, followed by 10 frames at 1 frame/min), immediately followed by a normal CT scan. The 25-min-delayed radioactivity intensity in the region of interest, i.e., the turbinate area, was used to evaluate the reabsorption rate of CSF circulation (see Fig. 5B). After scanning, the rats were euthanized and placed in the nuclear decay zone for radioactive decay. All procedures in this section were performed under radio-protection.

Likewise, the reabsorption efficiency of the turbinate area was assessed and visualized. The procedures were the same as those described above, with the only difference being the tracer injected, which was altered to 50 μl 1.5% Evans blue dye (Yuanye, Shanghai, China). After 25 min, the rat was euthanized, decapitated, and subjected to a median sagittal incision to examine the turbinate tissues under direct vision (see Fig. 5C). Images were captured using a Canon 7D camera.

### Western blot

The protein concentrations were quantified using the BCA method (Micro BCA protein Assay Reagent kit; Thermo Fisher Scientific Inc. Waltham, MA, USA), and 20 μg of protein were loaded into each lane of the western blot (protein immunoblot) for analysis of myostatin expression levels. Specifically, to assess myostatin expression, equal amounts of protein extracted from the RCDma and the RCDmi, the semispinalis capitis muscle and the gastrocnemius muscles obtained from the MSTN group (n = 3) and the CTL group (n = 3) were subjected to western blot. The rats were sacrificed two weeks after local injections, and their RCDma, RCDmi, and gastrocnemius muscles were extracted and frozen in liquid nitrogen. The anti-myostatin (GDF-8) was produced in goats (AF788, R&D systems, MN, USA), and the anti-α-tubulin was produced in rabbits (KG22771, KeyGEN BioTECH Co., Beijing, China) and used as the primary antibodies. Furthermore, the secondary antibodies, HRP-conjugated anti-Goat IgG (GE Healthcare) and HRP-conjugated anti-Rabbit IgG (GE Healthcare), were diluted in TBS with Tween-20 (TBST) in 5% skim milk. The bands were detected using ECL prime (GE Healthcare), and the experimental protocol was performed according to the manufacturer's instructions.

### Histology and microscopy

Following conventional perfusion and fixation, tissue samples comprising suboccipital muscle tissue, occipital bone, atlas, axis, and semispinalis capitis muscle were decalcified with ethylenediaminetetraacetic acid disodium salt (Na_2_EDTA•2H_2_O). Thereafter, regular paraffin embedding was performed, and 8-μm thick sections were obtained using a rotary microtome (Leica Micro HM450; Leica Microsystems GmbH, Wetzlar, Germany). To verify the result of model building, Masson trichrome (aniline blue) stained sections were photographed using a Nikon NIS image system (Nikon Eclipse 80i, Nikon, Tokyo, Japan). Additionally, Masson trichrome (light green) staining was also performed on RCDma and RCDmi to measure myocyte cross-section area and evaluate muscle condition after local injection. The results of the staining were quantified using Image-J software (National Institutes of Health). At least 100 myocytes were measured per animal to assess myocyte condition.

### Immunohistochemistry

In this study, two weeks after surgery, rats were euthanized and their brains were perfused with 4% paraformaldehyde. Choroid plexus in lateral ventricles were prepared for immunohistochemistry (IHC) after regular paraffin embedding. The primary antibodies used were AQP-1 mouse monoclonal antibody from Santa Cruz Biotechnology (1:500), AQP-4 mouse monoclonal antibody from Bioss (1:200), OTX-2 mouse monoclonal antibody from Santa Cruz Biotechnology (1:500), TTR sheep polyclonal antibody from Santa Cruz Biotechnology (1:400), KCNE-2 rabbit monoclonal antibody from Bioss (1:200), and Na + -k + -ATPase rabbit monoclonal antibody from Signalway Antibody (1:400). The incubation with the primary antibodies occurred for 18 h at 4 ℃ according to the product manual, and the negative control group used PBS instead of the primary antibody. Visualization was performed with HRP-conjugated secondary antibodies (Biotin-labeled goat anti-rabbit IgG (ZSGB-BIO); Biotin-labeled goat anti-mouse IgG (ZSGB-BIO); Biotin-labeled rabbit anti-sheep IgG (Signalway Antibody) and DAB. All stained sections were photographed using a Nikon NIS image system (Nikon Eclipse 80i, Nikon, Tokyo, Japan), and the optical density was semi-quantified using Image-J software (N.I.H.)^48^.

### Statistics

Statistical analysis was carried out using Prism 8.0 (Graphpad Software) and Microsoft Excel Statistics Toolkit (Microsoft). Paired t-tests were applied. Results shown are mean + SEM, a p-value of < 0.05 was statistically significant.

## Results

### Local injection of ACE-031 and BTX-A, as well as severance operation resulted in muscle status changes of RCDma and RCDmi

The study measured weight, food intake, and water intake before and 2 weeks after model establishment for each group of rats (Fig. 1A). There was no significant difference observed among the four groups of pre-operation rats. However, after modelling, only the SEV group showed significant differences in weight, food intake, and water intake relative to the CTL group, which could be attributed to the severance injury of suboccipital muscles. These results indicated that the basic physiological condition background among groups was relatively consistent, and no significant effect would give rise to the results of subsequent experiments.

**Figure 2:**
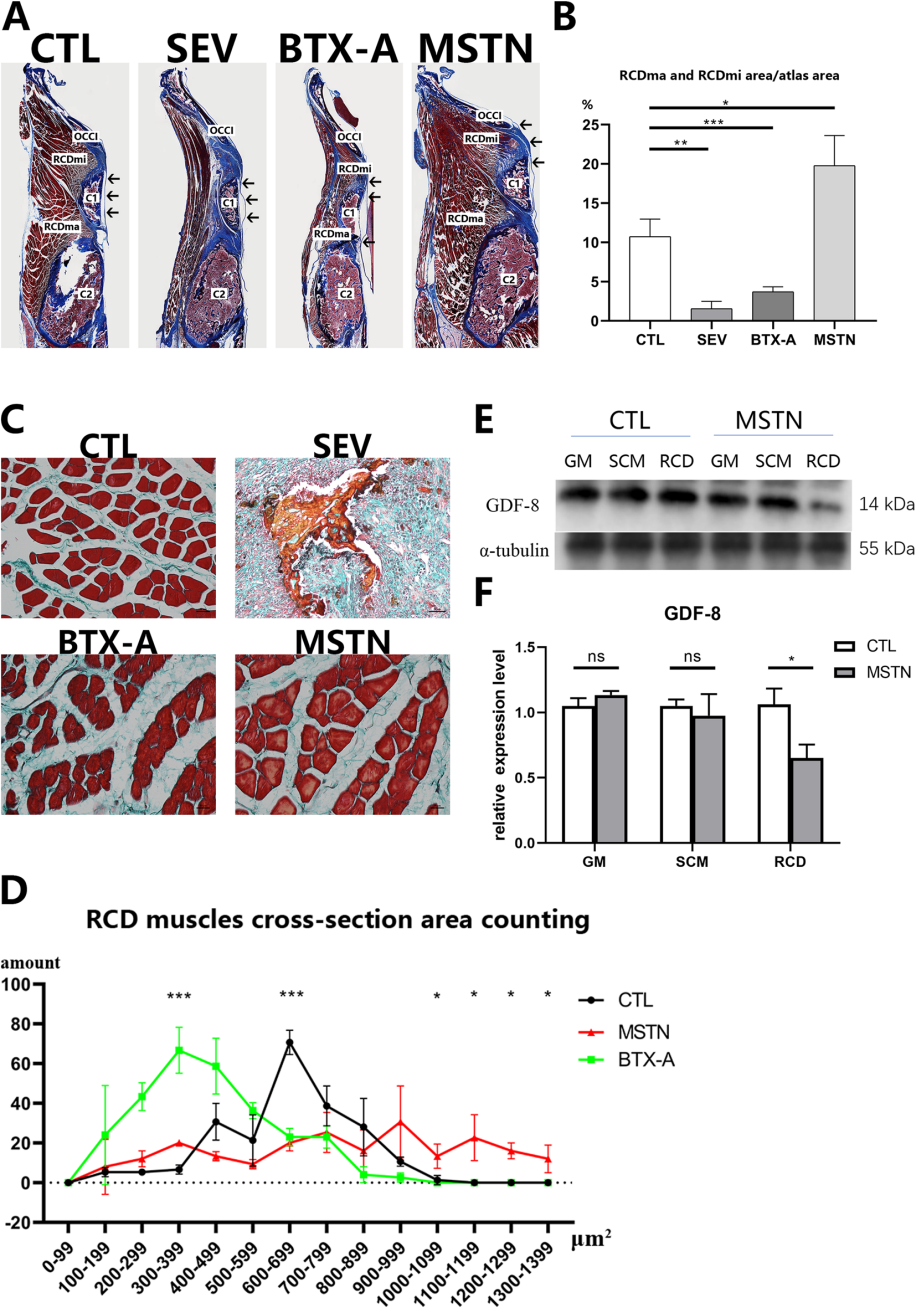
Verification of modeling results of all groups. (**A**) sagittal section of rats suboccipital area with Masson stain. (**B**) comparison of suboccipital muscle (RCDma and RCDmi) area/atlas area ratio. (**C**) representative cross-sections of RCDma muscle with Masson stain. (**D**) quantity statistics of cross-sectional area in different ranges of the RCDma and RCDmi muscles among CTL, BTX-A, and MSTN groups. (**E**) expression level of myostatin (GDF-8) in the gastrocnemius muscle, splenius capitis muscle, and RCDma and RCDmi muscles in CTL and MSTN groups rats. original gels are presented in Supplementary Fig. 1. (**F**) protein quantitative analysis of expression level of myostatin (GDF-8) in the gastrocnemius muscle, splenius capitis muscle, and RCDma and RCDmi muscles in CTL and MSTN groups rats. CTL, control group; SEV, severance group; BTX-A, BTX-A local injection group; MSTN, ACE-031 local injection group; GM, gastrocnemius muscle; SCM, splenius capitis muscle; RCD, rectus capitis dorsalis muscles; *, *P* < 0.05; **, *P* < 0.01; ***, *P* < 0.001; ns, no significance.

The study established suboccipital muscle hyperplasia and atrophy models after a single local injection of ACE-031 and BTX-A for 2 weeks. Western blot analysis was utilized to measure the levels of Myostatin (GDF-8) of RCDma and RCDmi since ACE-031 targets Myostatin (GDF-8). Gastrocnemius and splenius capitis muscle were used as controls (Fig. 2E). The study observed an obvious inhibition of GDF-8 gene in RCDma and RCDmi, with no level change in gastrocnemius and splenius capitis muscle (Fig. 2F).

Histological analysis of all four groups was conducted to verify the results of modelling. Relative to the CTL group, parasagittal sections of Masson trichrome (aniline blue) staining and analysis of muscle-atlas area ratio showed increased and decreased suboccipital muscle area in BTX-A group and MSTN group, respectively. Meanwhile, hardly any suboccipital muscles were observed in the SEV group (Fig. 2A,B). To semi-quantitatively estimate the hyperplasia, atrophy, and severance effect, Masson trichrome (light green) staining of RCDma and RCDmi on the cross section was performed (Fig. 2C). The amount of muscle fibers cross-sectional area was counted, and according to the mode, the results showed an increase and a decrease of the suboccipital muscle cross-section area in MSTN and BTX-A group, respectively (Fig. 2D). No obvious muscle tissue in the SEV group was observed. All these results showed that the established models of suboccipital muscle hyperplasia, atrophy, and severance were credible.

### The alteration of CSF secretion rate

**Figure 3:**
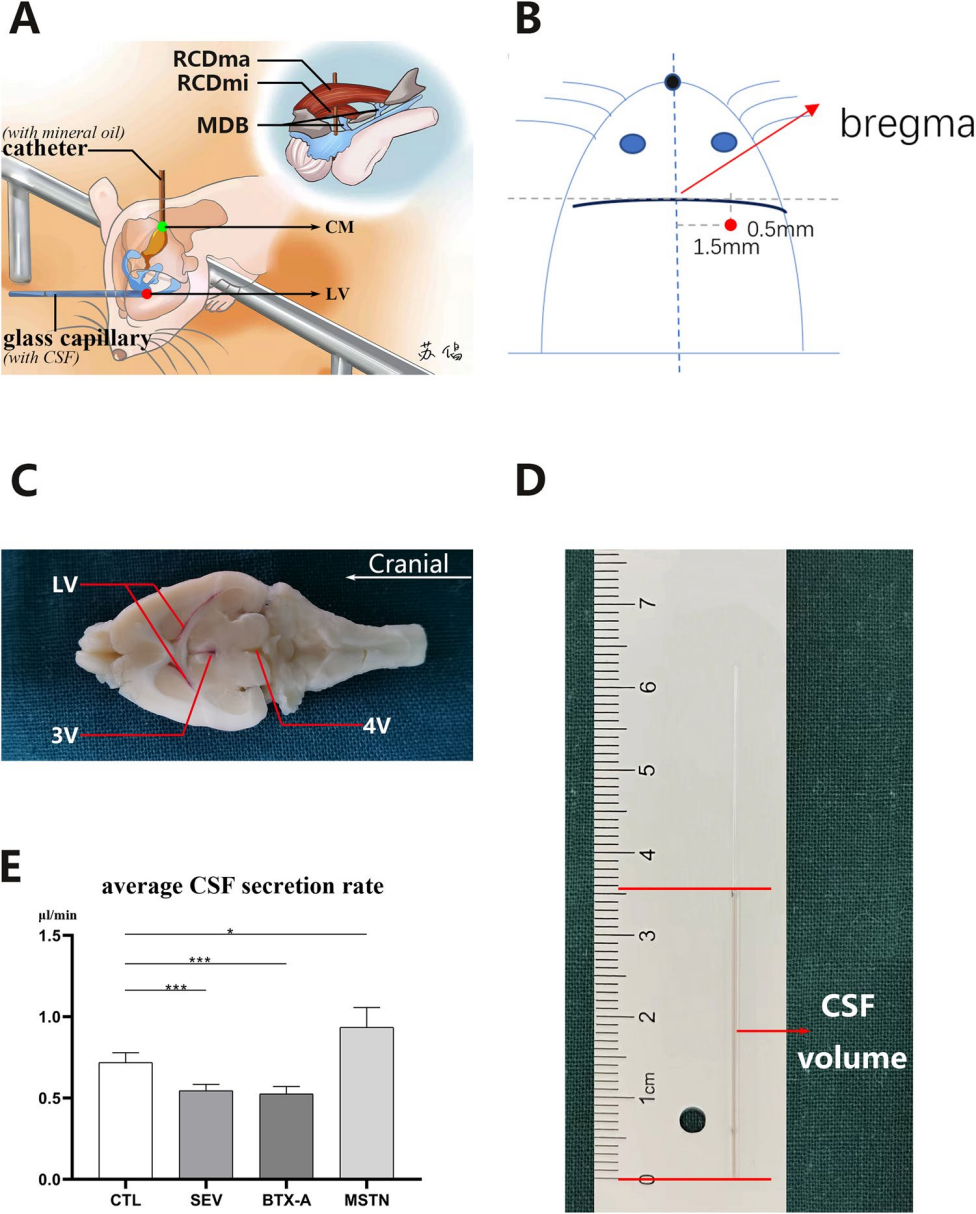
Suboccipital muscles interventions resulted in changes of CSF secretion rate. (**A**) schematic diagram of CSF secretion rate measurement method. (**B**) schematic diagram of the drilling site location. (**C**) eosin blockage testing result to verity the blockage efficiency of mineral oil. (**D**) representative diagram of CSF secretion rate measurement results. (**E**) statistical analysis of CSF secretion rate 2 weeks after modeling among all groups. RCDma: rectus capitis dorsalis major; RCDmi: rectus capitis posterior minor; MDB, myodural bridge; LV, lateral ventricle; 3 V, third ventricle; 4 V, fourth ventricle; CTL, control group; SEV, severance group; BTX-A, BTX-A local injection group; MSTN, ACE-031 local injection group; CM, cisterna magna; *, *P* < 0.05; **, *P* < 0.01; ***, *P* < 0.001; ns, no significance; , catheter insertion site; , glass capillary insertion site.

The potential impact of the MDB on CSF dynamics, mediated by the displacement of suboccipital muscles to the cervical SDM, has been investigated. The alteration of CSF secretion rate was estimated as a means of exploring this impact. The feasibility of the experimental method was verified through a blockage test, which involved the staining of ventricular walls with eosin following the blockage of the aqueduct of Sylvius with mineral oil. No leakage of CSF was observed, as evidenced by the absence of red staining in the fourth ventricle (Fig. 3C). The CSF secretion rate of all four groups was then calculated, with results demonstrating that the rate was 0.711 ± 0.05 µL/min (mean ± SD) in the CTL group, 0.545 ± 0.06 µL/min in the SEV group, 0.526 ± 0.07 µL/min in the BTX-A group, and 0.935 ± 0.1 µL/min in the MSTN group. Statistical analysis indicated that the CSF secretion rate was significantly increased in the MSTN group (*p* < 0.05), while it was significantly decreased in both the BTX-A group (*p* < 0.001) and the SEV group (*p* < 0.001) (Fig. 3E).

**Figure 4:**
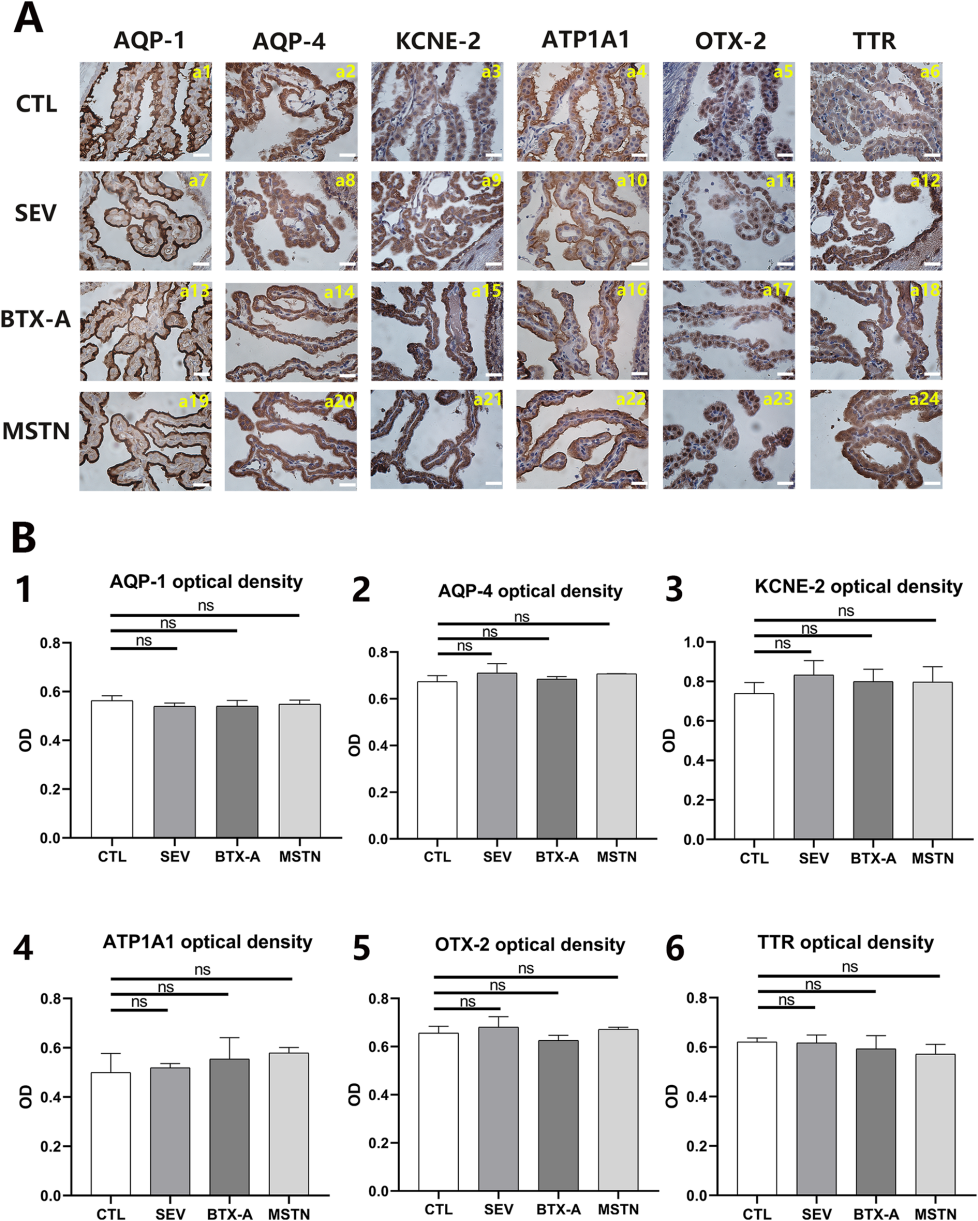
Suboccipital muscles interventions caused no significant change of examined CP-CSF-related proteins. (**A**) IHC results of AQP-1, AQP-4, KCNE-2, ATP1A1, OTX-2, and TTR 2 weeks after modeling among all groups. (**B**) statistical analysis of AQP-1 optical density. Scale bar: 20 μm. B. statistical analysis of AQP-4 optical density. (**C**) statistical analysis of KCNE-2 optical density. (**D**) statistical analysis of ATP1A1 optical density. (**E**) statistical analysis of OTX-2 optical density. (**F**) statistical analysis of TTR optical density. ns, no significance. CTL, control group; SEV, severance group; BTX-A, BTX-A local injection group; MSTN, ACE-031 local injection group; ns, no significance.

To further understand the mechanism of CSF secretion rate change, six CP-CSF-related proteins were selected for IHC semi-quantitative analysis. However, no significant difference in the protein levels of these modeled animals was observed in any group (Fig. 4A,B). These findings suggest that the effect of MDB on CSF dynamics is not mediated by changes in the levels of these selected proteins. Further studies are required to elucidate the underlying mechanisms involved.

### The change of CSF reabsorption rate

**Figure 5:**
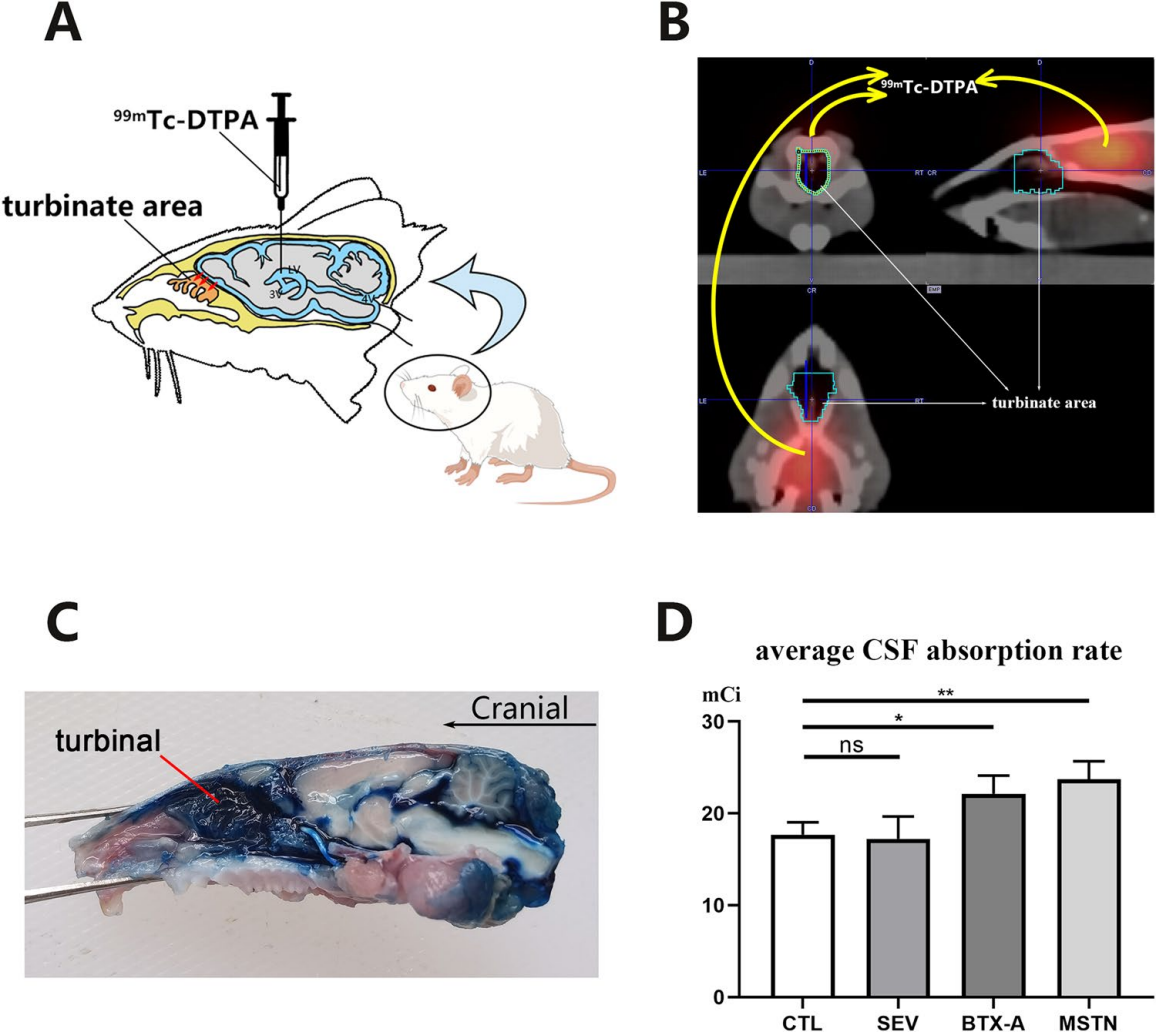
Suboccipital muscles interventions resulted in changes of CSF absorption rate at the turbinate area. (**A**) schematic diagram of lateral ventricle radionuclide injection and CSF absorption at the turbinate area. (**B**) schematic diagram of turbinate area delineation and radioactive intensity measurement after SPECT/CT scanning. (**C**) verification of CSF absorption efficiency of the turbinate area with Evans blue dye. (**D**) statistical analysis of CSF absorption rate 2 weeks after modeling among all groups. CTL, control group; SEV, severance group; BTX-A, BTX-A local injection group; MSTN, ACE-031 local injection group; *, *P* < 0.05; **, *P* < 0.01; ns, no significance; ← , the absorption of CSF at the turbinate area.

In addition to its potential impact on CSF secretion rate, MDB may also affect CSF dynamics through its influence on CSF reabsorption. To investigate this possibility, we measured CSF reabsorption rate in all experimental groups of rats. To confirm and visualize the efficiency of reabsorption in the turbinate area, we first conducted an experiment in which Evans blue was injected into an right-side lateral ventricle as a tracer. After 25 min, the majority of tracer was found to have accumulated in the turbinate area (Fig. 5C), indicating this as the primary site of CSF reabsorption. We then measured CSF reabsorption rate using 99mTc-DTPA and merged the image with a normal CT scanning image for positioning. The change in nuclide radioactivity 25 min after injection served as an indicator of CSF reabsorption rate. Our results demonstrated that, compared to the CTL group (18.65 ± 2.1 mCi, mean ± SD), both the BTX-A group (22.15 ± 2.1 mCi, *p* < 0.05) and the MSTN group (23.71 ± 1.9 mCi, *p* < 0.01) showed a significant increase in CSF reabsorption rate, while the SEV group (17.23 ± 2.0 mCi, *p* > 0.05) did not show a significant change (Fig. 5D).

These findings suggest that MDB may play a role in regulating CSF reabsorption, with both BTX-A and MSTN potentially enhancing this process. Further studies are needed to fully elucidate the mechanisms underlying these effects and their potential clinical relevance.

## Discussion

### MDBC affected CSF dynamics through contraction force changes of suboccipital musculature

**Figure 6:**
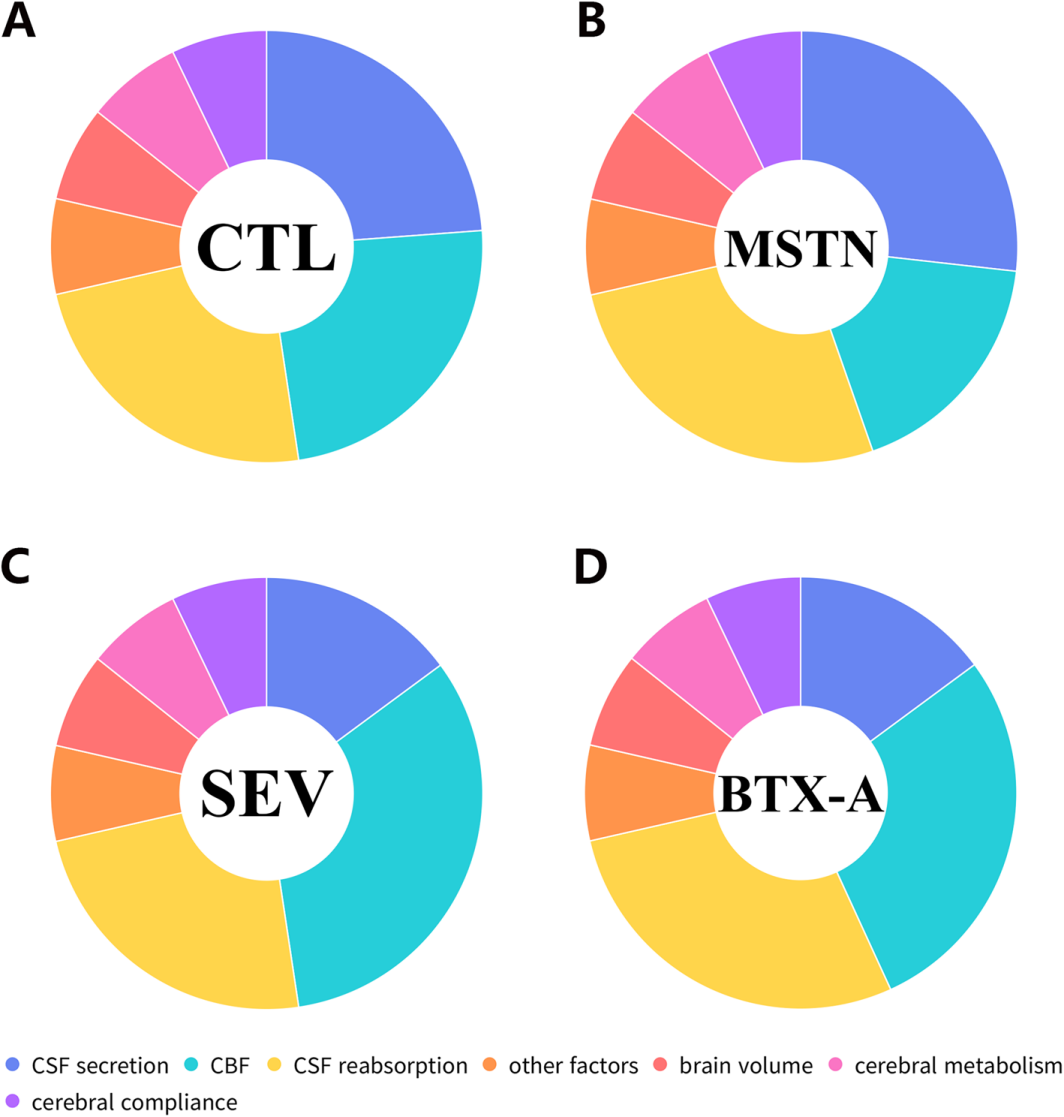
Schematic diagram of the dynamic balance of the intracranial environment. The whole circle represents intracranial environmental homeostasis. (**A**) Hypothetical intracranial homeostasis of rats in CTL group. Under physiological conditions, CSF secretion was considered to be balanced with CSF reabsorption. (**B**) Hypothetical intracranial homeostasis of rats in MSTN group, showing increased CSF secretion and reabsorption but decreased CBF. (**C**) Hypothetical intracranial homeostasis of rats in SEV group, showing decreased CSF secretion and increased CBF. (**D**) Hypothetical intracranial homeostasis of rats in BTX-A group, showing decreased CSF secretion but increased CSF reabsorption and CBF. The components represented by each color are shown in the legend. CTL, control group; SEV, severance group; BTX-A, BTX-A local injection group; MSTN, ACE-031 local injection group; CBF, cerebral blood flow.

According to the classic theory of CSF dynamics, the hydrostatic pressure gradient between CSF secretion and reabsorption sites is considered the fundamental driving force of CSF circulation^49^. This driving force offers a basic direction of CSF flow from high-pressure regions, where CSF forms, to low-pressure regions, where CSF reabsorbs^1^. In circumstances of intracranial homeostasis, the volume of CSF and intracranial blood flow reach a relative stable status until new changes occur (Fig. 6A). Among all the intracranial constituents, CSF and blood flow are the most adjustable components that perform quick responses to alterations of intracranial homeostasis^50–52^. Previous studies have identified the muscles directly attached to the dura mater as one of the power sources of CSF circulation^14,23,26^. The functional unit, referred to as the MDBC, affects CSF circulation in different ways.

The contraction force of suboccipital muscles can transmit to the dura mater, causing a displacement of the dura mater and leading to a regional negative pressure of the subarachnoid space, resulting in alterations of CSF flow. This effect may initially be considered local, but with constant stimulation, it could become global^27^. In addition to the speculation of dura mater displacement, MDBC is also thought to affect CSF dynamics by influencing cerebral blood flow (CBF). The suboccipital region is one of the most complex regions in humans, containing unique bony structures, several pairs of short muscles, abundant nerves, and vasculature^53,54^. These rich vascular communications correlate the movement of the suboccipital muscles with the regulation of intracranial blood flow^52,55^. Considering about the kinetoterapy of lower limbs varicosity, constant muscular relaxation and contraction circles may act as a pump to the local veins, propels the compression of the intramuscular and deep veins, which increase the venous pressure and promote the blood in the deep vein back to the heart^56^. Therefore, predictively, changes in the contraction force of suboccipital musculature alter the pressure on the wall of blood vessels, resulting in changes in the diameter of blood vessels that can affect the secretion and reabsorption of CSF. Quantification of these changes may be represented by changes in the volume of CSF and CBF. However, this predictive implication lacks of experimental support in the present study.

To further study the role of MDBC in CSF dynamics, we applied local injections of BTX-A and ACE-031 to functionally downregulate and upregulate the target muscles, respectively. BTX-A is a neurotoxin that binds to receptors on presynaptic cholinergic nerve terminals, blocking the release of acetylcholine^29^. Intramuscular injections of BTX-A can relieve focal spasticity and dystonia, and long-term injections of BTX-A can cause disuse atrophy of muscle^30,31^. ACE-031 is a specific inhibitor of myostatin (GDF-8) that can upregulate muscle size, mass, and strength^33,34^. Additionally, we severed the target muscles as a functional negative control and set another group of wild-type control with no operation.

The present study aimed to investigate the effects of suboccipital musculature on CSF dynamics in living rats. To this end, we measured the rate of CSF secretion, referred to as V_f_, in vivo and calculated CSF reabsorption rate, referred to as V_a_, by detecting the radioactive intensity of the turbinate area, which is known to be the main site of CSF reabsorption in rodents. We then examined changes in V_f_ and V_a_ under different conditions of suboccipital musculature.

According to previous studies, rats with ACE-031 blockage showed increased muscle strength^27^, whereas botulinum toxin reduced the muscle strength^57^; in the present study, rats in the MSTN group exhibited increased muscle mass in the right and left cranial dorsal muscles (RCDma and RCDmi), along with increased V_f_ and V_a_. Conversely, rats in the BTX-A group displayed decreased muscle mass in RCDma and RCDmi, as well as decreased V_f_ but increased V_a_. As a functional negative control, rats in the SEV group exhibited rare RCDma and RCDmi muscles, and showed a significant decrease in V_f_ but only a slight decrease in V_a_ with no statistical significance. These findings suggest that changes in suboccipital musculature can significantly affect CSF dynamics, as measured by V_f_ and V_a_.

### Hyperplasia and severance of suboccipital musculature may lead to intracranial homeostasis changes by altering V_f_ and V_a_

As stated above, CSF is one of the most adjustable components and plays an important role in intracranial homeostasis. For myostatin blockage, our previous^27^ and the present studies collectively indicated that muscle hyperplasia brought increased contraction force which MDB transmitted; Moreover, since MDB directly link to the SDM and has been acknowledged as a fixing device of SDM^22^, it is highly predictive that the suboccipital hyperplasia could increase the displacement of SDM pulled by MDB. Stated another word, there is a great potential that the function of the MDBC unit was enhanced. In this case, we at the same time measured an increase in both V_f_ and V_a_. According to these results, we speculated that enhanced MDBC function may lead to increased V_f_. Assuming no other change occurred, CSF volume would increase for the formation speed exceeded the reabsorption speed. As a result, the hydrostatic pressure gradient between CSF formation sites and CSF reabsorption sites increased, which offered a greater driving force for CSF reabsorption. Thus, V_a_ increased as well to reach, or tend to reach, an intracranial homeostasis. Therefore, to keep a relatively stable intracranial environment and maintain the CSF volume, an increased V_a_ was observed after the increased V_f_ (Fig. 6B).

For SEV group, the remove of RCDma and RCDmi caused declined MDBC function from the view point of SDM pulling. So compared with MSTN group, V_f_ of SEV group decreased. When V_f_ decreased, CSF formation speed receded CSF reabsorption speed, leading to reduced CSF volume and decreased hydrostatic pressure gradient. In terms of the fundamental driving forces, it seemed to generate a decreased V_a_. But in present study, although presenting a slightly decline trend, V_a_ of SEV group was observed no difference with CTL group. So, we speculated that MDBC may play a significant role in V_a_ feedback and regulation. In SEV group, loss of RCDma and RCDmi made the function unit MDBC incomplete, which may lead to reduced susceptibility of CSF reabsorption regulation. Thus, rats in SEV group lost their ability of V_a_ regulation in response to CSF volume and hydrostatic pressure gradient changes. At the same time, local fibrous scar repair limited the adjustability of vessel diameter, making CBF at a high level. Thus, the intracranial environment maintained relatively stable although CSF volume may be decreasing (Fig. 6C).

In summary, the results of the present study indicate that changes in the suboccipital musculature can affect both CSF formation and reabsorption rates, which can have significant implications for intracranial homeostasis. The increased muscle mass and strength observed in the MSTN group resulted in enhanced MDBC function, leading to increased CSF volume and hydrostatic pressure gradient, and ultimately increased CSF reabsorption rate. On the other hand, the loss of RCDma and RCDmi in the SEV group resulted in decreased MDBC function, leading to decreased CSF volume and hydrostatic pressure gradient, and a potential reduction in the ability to regulate CSF reabsorption rate in response to changes in CSF volume and pressure. Despite these changes, the intracranial environment was supposed to maintain relatively stable in both groups due to compensatory mechanisms such as vessel diameter adjustment and CBF regulation. These findings may have important implications for understanding the pathophysiology of disorders such as hydrocephalus and intracranial hypertension, as well as potential therapeutic interventions targeting the suboccipital musculature.

### Atrophy, compared with hyperplasia, of suboccipital musculature led to not exactly opposite intracranial homeostasis changes by altering V_f_ and V_a_.

In the present study, we investigated the effects of suboccipital musculature alterations on intracranial homeostasis by inducing atrophy and hyperplasia in animal models. Specifically, we observed that in the BTX-A group (Fig. 6D), which exhibited declined function of the MDBC unit due to muscle atrophy and decreased muscle strength, V_f_ decreased, leading to less and slower formation of CSF, and subsequently, reduced CSF volume. This decline in CSF formation would usually lead to a reduction or tend to reduce CSF reabsorption; however, we measured an increase in V_a_ in the BTX-A group, which may lead to further reduction of CSF volume. Although the exact mechanisms underlying this phenomenon remain to be confirmed, we proposed two possible speculations.

First, we speculated that CBF plays a crucial role in this regulatory process. Due to muscle atrophy, the contraction force of suboccipital muscles is reduced, leading to a decrease in the pressure they produce on the blood vessels in the suboccipital region. This decrease in pressure results in a relative increase in vessel diameter, thereby maintaining CBF at a relatively high level to compensate for intracranial volume changes.

Second, we speculated that suboccipital musculature may be critical for V_a_ regulation. Despite the status of muscles, significant changes in V_a_ were observed in both the MSTN and the BTX-A groups, whereas barely any change was observed in the SEV group, in which the RCDma and RCDmi were removed. This suggests that suboccipital musculature may contribute to the reaction to CSF volume or reabsorption changes.

It should be noted that the experiments were conducted at a time point of 2 weeks after modelling, which corresponded to the clinical course. This may not be long enough to reach a relatively stable status, and the V_a_ increase observed in the BTX-A group could be a reactive change of MDBC status alteration at this time point. Further experiments are required to confirm the exact mechanisms underlying these observations.

### Implication on humans

The MDB is recognized as a direct connective linkage of suboccipital muscles and the cervical dura mater. In middle 2023, a novel anatomical definition of occipito-atlantal cistern (OAC) has been introduced, OAC is a subarachnoid space independent from the cisterna magna. More to the point, the dura mater which in the posterior of OAC is just where the MDB connects on humans^58^. According to the present study, it is indicative that the CSF production and absorption correlates with dysfunction of suboccipital muscles. More recently, our team found OAC also exist in rats (data not shown). Additionally, considering the MDB universally exist in mammals^20^, these collectively suggested that there may be strong potential between MDBC and clinical CSF disorders, no matter in human or other mammals. During the present study, the experimental animals shared highly similar patterns of life and daily routines, which includes similar food and water intakes, similar sleep regulations, similar exercise intensities, etc. In addition, considering the animals with or without surgical treatments exhibited similar food/water intake, body weight. These in all indicated that these animals shared similar normal physiologies, which includes non-hindered body movements. Consequently, although these animals exhibited V_a_ and V_f_ changes, the inductive factors were easy to track.

However, humans feature different life regulations and schedules, different dietary habits, and different enthusiasm to exercise; not to mention the humans spend more than half their lives in upright position. These collectively result in different homeostasis of circulation, respiratory, as well as endocrine system, which are all important affecters of CSF dynamics. Furthermore, in human the aqueduct is compared to quadrupeds oriented in a more vertical position and the requirement to equilibrate CSF against hydrostatic forces is much higher than in quadrupeds.

Nevertheless, humans spend nearly halfway of their lives in laying down or sitting positions, and these are very similar to the conditions of the modeling animals in the present study. The MDBC could be a key regulator while in sleeping or resting conditions for humans. As this result, long-time improper postures and excessive burden to the neck have potentials leading to CSF disorders; conversely, it is also inferred that a proper exercise which aim to keep or rebuild the functions of neck muscle (especially suboccipital muscles) could be an effective way for the prevention and therapy to the diseases which related to CSF dynamics disorders. On the other hand, it also provided a mechanical support for the efficacy of cervical massage and manipulative therapy.

### Meaning and limitation

The presence of MDB in suboccipital muscles establishes a unique anatomical connection between these muscles and the dura mater^16^. This study investigated the impact of changes in MDBC on the fundamental components of CSF dynamics, namely V_f_ and V_a_, and their potential influence on intracranial homeostasis. Our results provide direct experimental evidence supporting the idea that MDBC can affect CSF circulation, as we observed changes in V_f_ and V_a_ following intervention in suboccipital muscles. We used a 2-week animal model to mimic the course of the clinical patient, which is more relevant than immediate effects assessed in previous experiments.

However, there are some limitations to our study. Firstly, the measurement method chosen for this experiment differs from previous perfusion methods and may not fully represent actual V_f_ and V_a_ values^59^. Fluid (both the oil-blockage of aqueduct and tracer injection into the lateral ventricles) may affect the real-time physiological CSF dynamics. However so far, due to the technical limitations, there is no perfect non-invasive measuring method of CSF production/absorption rates^60^. A novel non-invasive method was firstly described in 2020 to assess human the water transition of the blood-cerebrospinal fluid barrier^61^, this study certainly showed us a novel perspective to assess the CSF production rate non-invasively, and yet not 100% reliable because of the different study focus. In the present study, the possible alterations of CSF dynamics caused by fluid injections had been reduced as much as possible by controlling the experimental background as consistent as possible. Nevertheless, our corresponding speculations based on these results remain valid. Secondly, the present study simplified the model of CSF dynamics by observing only V_f_ and V_a_ and speculating the possible changes in intracranial homeostasis. We did not investigate diseases or symptoms associated with altered suboccipital muscle status, such as headache, which is closely linked to CSF and intracranial pressure^62–65^. Third, limited to the small volume of the suboccipital muscles, we did not find any direct way to test the contractive forces of the RCDma and RCDmi, although our previous study and publication of other researchers evidently demonstrated the correlations of contraction force changes with muscular atrophy and hyperplasia, a direct experimental proof remains unpresented. Fourth, for the muscular atrophy/hyperplasia models, the implications on related blood vessels have not yet been test, and thus the experimental results regarding V_a_ and V_F_ changes have not yet been fully interpreted. Moreover, the 2-week modelling duration used in this study may not fully correspond to the clinical course of cervicogenic headaches, considering most of headaches present chronicy^64,65^.

In conclusion, our study provides evidence supporting the hypothesis that MDBC can affect CSF circulation and intracranial homeostasis through changes in V_f_ and V_a_. Future studies should focus on the relationship between MDBC and CSF dynamics in greater depth, investigating potential clinical outcomes of altered suboccipital muscle status, including headache, and accounting for the complexity introduced by the perivascular spaces and the glymphatic system.

### Supplementary Information

Supplementary Information.

## Data Availability

Within the manuscript and its Supporting Information files.

## References

[1] Sakka L, Coll G, Chazal J (2011). Anatomy and physiology of cerebrospinal fluid. Eur. Ann. Otorhinolaryngol. Head Neck Dis..

[2] Tumani H, Huss A, Bachhuber F (2017). The cerebrospinal fluid and barriers - anatomic and physiologic considerations. Handb. Clin. Neurol..

[3] Spector R, Keep RF, Robert Snodgrass S, Smith QR, Johanson CE (2015). A balanced view of choroid plexus structure and function: Focus on adult humans. Exp. Neurol..

[4] Capel C (2014). Insights into cerebrospinal fluid and cerebral blood flows in infants and young children. J. Child Neurol..

[5] Bothwell SW, Janigro D, Patabendige A (2019). Cerebrospinal fluid dynamics and intracranial pressure elevation in neurological diseases. Fluids and Barriers CNS.

[6] Fame RM, Cortés-Campos C, Sive HL (2020). Brain ventricular system and cerebrospinal fluid development and function: light at the end of the tube: a primer with latest insights. BioEssays: News Rev. Mol. Cell. Dev. Biol..

[7] Baselli G (2022). Real-time phase-contrast MRI to monitor cervical blood and cerebrospinal fluid flow beat-by-beat variability. Biosensors (Basel).

[8] Langner S (2017). Diagnosis and differential diagnosis of hydrocephalus in adults. Rofo.

[9] Czosnyka M, Czosnyka Z, Momjian S, Pickard JD (2004). Cerebrospinal fluid dynamics. Physiol. Meas..

[10] Hirasawa M, de Lange ECM (2022). Revisiting cerebrospinal fluid flow direction and rate in physiologically based pharmacokinetic model. Pharmaceutics.

[11] Zhu DC, Xenos M, Linninger AA, Penn RD (2006). Dynamics of lateral ventricle and cerebrospinal fluid in normal and hydrocephalic brains. J. Mag. Res. Imag.: JMRI.

[12] Friese S, Hamhaber U, Erb M, Kueker W, Klose U (2004). The influence of pulse and respiration on spinal cerebrospinal fluid pulsation. Invest. Radiol..

[13] Muccio M (2021). Upright versus supine MRI: effects of body position on craniocervical CSF flow. Fluids Barriers CNS.

[14] Xu Q (2016). Head movement, an important contributor to human cerebrospinal fluid circulation. Sci. Rep..

[15] Hack GD, Koritzer RT, Robinson WL, Hallgren RC, Greenman PE (1995). Anatomic relation between the rectus capitis posterior minor muscle and the dura mater. Spine.

[16] Zheng N (2018). Orientation and property of fibers of the myodural bridge in humans. Spine J.: Off. J. North Am. Spine Soc..

[17] Liu P (2018). The myodural bridges' existence in the sperm whale. PLoS One.

[18] Okoye CS, Zheng N, Yu SB, Sui HJ (2018). The myodural bridge in the common rock pigeon (Columbia livia): Morphology and possible physiological implications. J. Morphol..

[19] Zhang JH (2016). Connection of the posterior occipital muscle and dura mater of the siamese crocodile. Anat. Rec. (Hoboken).

[20] Zheng N (2017). The universal existence of myodural bridge in mammals: an indication of a necessary function. Sci. Rep..

[21] Scali F, Marsili ES, Pontell ME (2011). Anatomical connection between the rectus capitis posterior major and the dura mater. Spine.

[22] Scali F, Pontell ME, Enix DE, Marshall E (2013). Histological analysis of the rectus capitis posterior major's myodural bridge. Spine J.: Off. J. North Am. Spine Soc..

[23] Ma Y (2021). The morphology, biomechanics, and physiological function of the suboccipital myodural connections. Sci. Rep..

[24] Zheng N (2020). The myodural bridge complex defined as a new functional structure. Surg. Radiol. Anat.: SRA.

[25] Young BA, Greer S, Cramberg M (2021). Slithering CSF: cerebrospinal fluid dynamics in the stationary and moving viper boa candoia aspera. Biology.

[26] Xu Q (2021). Head-nodding: a driving force for the circulation of cerebrospinal fluid. Sci. Rep..

[27] Li C (2022). The relationship between myodural bridges, hyperplasia of the suboccipital musculature, and intracranial pressure. PLoS One.

[28] Pinna G, Alessandrini F, Alfieri A, Rossi M, Bricolo A (2000). Cerebrospinal fluid flow dynamics study in Chiari I malformation: implications for syrinx formation. Neurosurg. Focus.

[29] Durand PD (2016). Botulinum toxin and muscle atrophy: a wanted or unwanted effect. Aesthet. Surg. J..

[30] Nassif, A. D., Boggio, R. F., Espicalsky, S. & Faria, G. E. L. High Precision Use of Botulinum Toxin Type A (BONT-A) in Aesthetics Based on Muscle Atrophy, Is Muscular Architecture Reprogramming a Possibility? A Systematic Review of Literature on Muscle Atrophy after BoNT-A Injections. *Toxins (Basel)* **14**, 10.3390/toxins14020081 (2022). 10.3390/toxins14020081 PMC8878196 35202109

[31] Xu J (2020). Knee muscle atrophy is a risk factor for development of knee osteoarthritis in a rat model. J. Ortho. Trans..

[32] Gonzalez-Cadavid NF (1998). Organization of the human myostatin gene and expression in healthy men and HIV-infected men with muscle wasting. Proc. Natl. Acad. Sci. United States Am..

[33] Tsuchida K (2008). Targeting myostatin for therapies against muscle-wasting disorders. Curr. Opin. Drug Discov. Devel..

[34] McPherron AC, Lawler AM, Lee SJ (1997). Regulation of skeletal muscle mass in mice by a new TGF-beta superfamily member. Nature.

[35] McPherron AC, Lee SJ (1997). Double muscling in cattle due to mutations in the myostatin gene. Proc. Natl. Acad. Sci. United States Am..

[36] Karimy JK (2015). A novel method to study cerebrospinal fluid dynamics in rats. J. Neurosci. Methods.

[37] Liu G (2020). Direct measurement of cerebrospinal fluid production in mice. Cell Rep..

[38] Damkier HH, Brown PD, Praetorius J (2013). Cerebrospinal fluid secretion by the choroid plexus. Physiol. Rev..

[39] Brown PD, Davies SL, Speake T, Millar ID (2004). Molecular mechanisms of cerebrospinal fluid production. Neuroscience.

[40] McCrossan, Z. A., Roepke, T. K., Lewis, A., Panaghie, G. & Abbott, G. W. Regulation of the Kv2.1 potassium channel by MinK and MiRP1. *J. Membr. Biol.* **228**, 1–14, 10.1007/s00232-009-9154-8 (2009). 10.1007/s00232-009-9154-8 PMC2849987 19219384

[41] Speake T, Freeman LJ, Brown PD (2003). Expression of aquaporin 1 and aquaporin 4 water channels in rat choroid plexus. Biochimica et biophysica acta.

[42] Uldall M, Botfield H, Jansen-Olesen I, Sinclair A, Jensen R (2017). Acetazolamide lowers intracranial pressure and modulates the cerebrospinal fluid secretion pathway in healthy rats. Neurosci. Lett..

[43] Spatazza J (2013). Choroid-plexus-derived Otx2 homeoprotein constrains adult cortical plasticity. Cell Rep..

[44] Chanoine JP (1992). Role of transthyretin in the transport of thyroxine from the blood to the choroid plexus, the cerebrospinal fluid, and the brain. Endocrinology.

[45] Maloveska M (2018). Dynamics of Evans blue clearance from cerebrospinal fluid into meningeal lymphatic vessels and deep cervical lymph nodes. Neurol. Res..

[46] Nagra, G. *et al.* Impaired lymphatic cerebrospinal fluid absorption in a rat model of kaolin-induced communicating hydrocephalus. *Am. J. Physiol. Regulat., Int. Comp. Physiol.* **294**, R1752–1759, 10.1152/ajpregu.00748.2007 (2008). 10.1152/ajpregu.00748.2007 18305019

[47] Nagra, G., Koh, L., Zakharov, A., Armstrong, D. & Johnston, M. Quantification of cerebrospinal fluid transport across the cribriform plate into lymphatics in rats. *Am. J. Physiol. Regulat., Int. Comp. Physiol.* **291**, R1383–1389, 10.1152/ajpregu.00235.2006 (2006). 10.1152/ajpregu.00235.2006 16793937

[48] Schindelin J (2012). Fiji: an open-source platform for biological-image analysis. Nat. Methods.

[49] Cottrell, J. & Cottrell, P. P. (Elsevier, 2017).

[50] Hoffmann J (2017). Impaired cerebrospinal fluid pressure. Handb. Clin. Neurol..

[51] Pinto, V. L., Tadi, P. & Adeyinka, A. in *StatPearls* (StatPearls Publishing Copyright © 2022, StatPearls Publishing LLC., 2022).

[52] Nowaczewska, M. & Kaźmierczak, H. Cerebral Blood Flow in Low Intracranial Pressure Headaches-What is Known? *Brain Sci.* **10**, 10.3390/brainsci10010002 (2019). 10.3390/brainsci10010002 PMC7016724 31861526

[53] George, T. & Tadi, P. in *StatPearls* (StatPearls Publishing, Copyright © 2023, StatPearls Publishing LLC., 2023).

[54] Standring, S. *Gray's anatomy e-book: the anatomical basis of clinical practice*. (Elsevier Health Sciences, 2021).

[55] Nathoo, N., Caris, E. C., Wiener, J. A. & Mendel, E. History of the vertebral venous plexus and the significant contributions of Breschet and Batson. *Neurosurgery* **69**, 1007–1014; discussion 1014, 10.1227/NEU.0b013e3182274865 (2011). 10.1227/NEU.0b013e3182274865 21654535

[56] Tew GA (2015). Supervised exercise training as an adjunctive therapy for venous leg ulcers: study protocol for a randomised controlled trial. Trials.

[57] Patel J, Cardoso JA, Mehta S (2019). A systematic review of botulinum toxin in the management of patients with temporomandibular disorders and bruxism. British Dent. J..

[58] Li YF (2023). A valuable subarachnoid space named the occipito-atlantal cistern. Sci. Rep..

[59] Artru, A. Cerebrospinal fluid. (1994).

[60] Liu G, Ladrón-de-Guevara A, Izhiman Y, Nedergaard M, Du T (2022). Measurements of cerebrospinal fluid production: a review of the limitations and advantages of current methodologies. Fluids Barriers CNS.

[61] Evans PG (2020). Non-Invasive MRI of Blood-Cerebrospinal Fluid Barrier Function. Nat. Commun..

[62] Grgić V (2007). Cervicogenic headache: etiopathogenesis, characteristics, diagnosis, differential diagnosis and therapy. Lijec Vjesn.

[63] Alix, M. E. & Bates, D. K. A proposed etiology of cervicogenic headache: the neurophysiologic basis and anatomic relationship between the dura mater and the rectus posterior capitis minor muscle. *J. Manipul. Physiol. Ther.* **22**, 534–539. 510.1016/S0161–4754(1099)70006–70000. 10.1016/S0161-4754(99)70006-0 10543584

[64] Cittadini E, Caso V (2012). Headache. Front Neurol. Neurosci..

[65] Zhu K, Born DW, Dilli E (2020). Secondary headache: current update. Headache.

