# Supplementary material for: The relationship between myodural bridge, atrophy and hyperplasia of the suboccipital musculature, and cerebrospinal fluid dynamics
Source: Sci Rep. 2023 Nov 2;13:18882. doi: 10.1038/s41598-023-45820-x (PMC10622500; doi:10.1038/s41598-023-45820-x)
Supplement: Supplementary file 1 — Supplementary Information. [file 41598_2023_45820_MOESM1_ESM.pdf]

Supplementary Figure 1

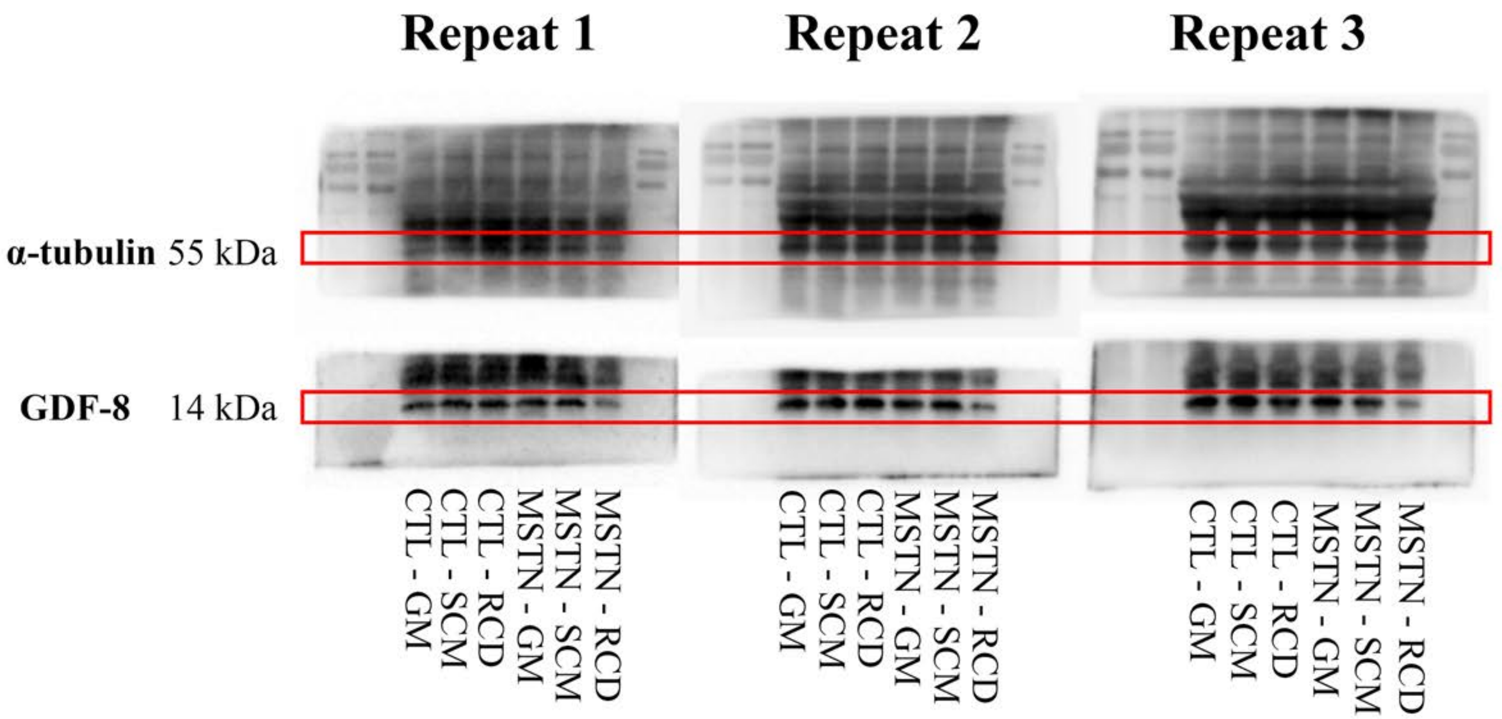

SF1-WB raw images: Original gel information for WesternBlot. Repeat 2 is the original gel information of Fig.2 E. Every repeat image shows a full-length gel, which was divided into two parts.
